# Multiple TGF-β Superfamily Signals Modulate the Adult *Drosophila* Immune Response

**DOI:** 10.1016/j.cub.2011.08.048

**Published:** 2011-10-11

**Authors:** Rebecca I. Clark, Katie J. Woodcock, Frédéric Geissmann, Céline Trouillet, Marc S. Dionne

**Affiliations:** 1 Centre for the Molecular and Cellular Biology of Inflammation and Peter Gorer Department of Immunobiology, King's College London School of Medicine, London SE1 1UL, UK

## Abstract

TGF-β superfamily signals play complex roles in regulation of tissue repair and inflammation in mammals [1]. *Drosophila melanogaster* is a well-established model for the study of innate immune function [2, 3] and wound healing [4–7]. Here, we explore the role and regulation of two TGF-β superfamily members, *dawdle* and *decapentaplegic* (*dpp*), in response to wounding and infection in adult *Drosophila*. We find that both TGF-β signals exhibit complex regulation in response to wounding and infection, each is expressed in a subset of phagocytes, and each inhibits a specific arm of the immune response. *dpp* is rapidly activated by wounds and represses the production of antimicrobial peptides; flies lacking *dpp* function display persistent, strong antimicrobial peptide expression after even a small wound. *dawdle*, in contrast, is activated by Gram-positive bacterial infection but repressed by Gram-negative infection or wounding; its role is to limit infection-induced melanization. Flies lacking *dawdle* function exhibit melanization even when uninfected. Together, these data imply a model in which the bone morphogenetic protein (BMP) *dpp* is an important inhibitor of inflammation following sterile injury whereas the activin-like *dawdle* determines the nature of the induced immune response.

## Graphical abstract

**Graphical Abstract:**

**Figure:**
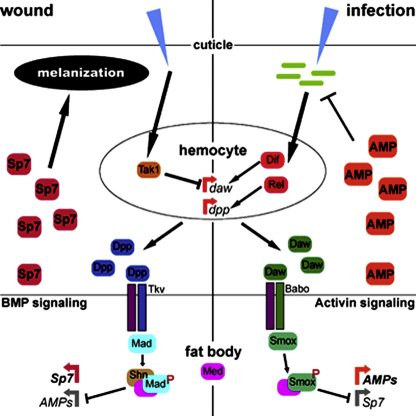

**Highlights:**

► BMP and activin signals respond differently to wound- or infection-induced cues ► BMP and activin signals repress different branches of the fly immune response ► These signals are expressed by subsets of hemocytes

## Results and Discussion

### *decapentaplegic* and *dawdle* Are Regulated by Immune Challenge

The innate immune response and its underlying pathways are highly conserved between *Drosophila* and mammals [3, 8, 9]. Although much work in *Drosophila* has focused on the central pathways of pattern recognition [3], many other signals modulate innate immune mechanisms, and many of these are also evolutionarily conserved [8, 9]. Because TGF-β superfamily signals are critical regulators of mammalian immune responses [1], we examined the immune regulation of two of these signals in the fly: *decapentaplegic* (*dpp*), a bone morphogenetic protein (BMP)-type signal, and *dawdle* (*daw*), an activin/TGF-β-like signal.

**Figure 1:**
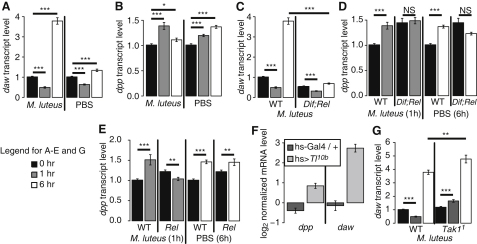
*dpp* and *daw* Are Regulated by Immune Challenge (A and B) *daw* and *dpp* expression in wild-type flies following *M. luteus* infection or PBS injection, normalized to untreated controls. Times given are the interval between treatment and sample collection. (C and D) *daw* and *dpp* expression following *M. luteus* infection in *Dif;Rel* double mutants (*Dif^2^ cn bw;Rel^e20^*) and wild-type controls (repeated from A and B). Expression is normalized to untreated wild-type controls. (E) *dpp* expression following *M. luteus* infection or PBS injection in *Rel* mutants (*Rel^e38^/Rel^e20^*) and wild-type controls (repeated from B). Expression is normalized to untreated wild-type controls. (F) *dpp* and *daw* expression 3 hr after heat shock in flies carrying UAS-*Tl^10b^* (*w;*UAS-*Tl^10b^.*myc/*tubulin-Gal80^ts^;hs-Gal4*/+) and driver-only controls (*w;tubulin-Gal80^ts^/+;hs-Gal4*/+). Expression after heat shock is normalized to non-heat-shocked genotype controls. (G) *daw* expression following *M. luteus* infection in *Tak1* mutants (*Tak1^1^*) and wild-type controls (repeated from A). Means are shown ±SEM. Assays were performed by qRT-PCR, and expression was normalized to *Rpl1*. ^∗∗∗^p < 0.001, ^∗∗^p < 0.01, ^∗^p < 0.05 by Mann-Whitney test. See also Figure S1.

*dpp* and *daw* expression were regulated by immune challenge. *daw* expression was initially repressed 1 hr after injection of either *Micrococcus luteus* or sterile phosphate-buffered saline (PBS) and subsequently induced 6 hr after *M. luteus* infection (Figure 1A). *dpp* was induced by either sterile wounding or infection, though with slightly different timing (Figure 1B). *E. coli* infection did not change *dpp* or *daw* expression beyond the effect of wounding alone (see Figure S1 available online).

To analyze the signaling underlying *daw* and *dpp* regulation by *M. luteus* infection, we first examined the role of NF-κB family members. In adult flies, the Toll pathway acts via the NF-κB-like factor *Dif*, while the imd pathway acts via the NF-κB-like factor *Rel* [3]. In *Dif;Rel* double mutants, baseline expression of *daw* was reduced and its induction by *M. luteus* infection was eliminated, but the early repression of *daw* was unaffected (Figure 1C). Untreated *Dif;Rel* mutants showed higher expression of *dpp* than controls, but, as with *daw*, induction of *dpp* by wounding or infection was lost (Figure 1D).

*dpp* and *daw* are therefore NF-κB-regulated. To assess the relative contributions of Toll and imd pathways, we assayed expression of *dpp* and *daw* in flies mutant only for *Rel*. Untreated *Rel* mutants showed increased baseline expression of *dpp* and *daw*, similar to *Dif;Rel* double mutants (Figure S1C; Figure 1E). Loss of *Rel* alone did not impair *daw* induction following infection (Figure S1C). However, the peak in *dpp* expression at 1 hr following *M. luteus* infection was lost in *Rel* mutants, though some *dpp* induction following wounding was retained (Figure 1E). To determine whether *Toll* signaling was sufficient to induce *dpp/daw*, we expressed activated *Toll* (UAS-*Tl*^10b^; [10]) with heat-shock *Gal4* (Figure S1D). Both *dpp* and *daw* were induced by *Toll* activation (Figure 1F). These data suggest that *Rel* drives *dpp* induction upon infection whereas *Dif* does so in response to wounding. In contrast, *Dif* drives *daw* expression upon infection.

Finally, we tested the role of the Jun N-terminal kinase (JNK) pathway in regulation of *dpp* and *daw* by wounds; this pathway is required for wound healing in *Drosophila* [4, 5]. Immune-induced JNK activation is mediated by *Tak1* downstream of *imd*, and in larvae *Tak1* is required for activation of JNK by sterile wounds [11, 12]. Therefore, we assayed *dpp* and *daw* in *Tak1* mutants. *Tak1* mutants showed a significant increase in baseline *dpp* expression relative to controls (Figure S1E). Loss of *Tak1* did not significantly alter *dpp* induction following infection or wounding (Figure S1E), but *daw* repression 1 hr following injection with PBS or *M. luteus* was abolished in *Tak1* mutants (data not shown; Figure 1G). Thus, *Tak1* activation represses *daw*.

These data indicate that JNK and NF-κB regulate *dpp* and *daw* in different immune contexts. The differences in regulation of these ligands suggested that they might play distinct roles in the immune response.

### *dpp* Suppresses the AMP Response to Wounding

We next examined the function of wound-induced Dpp. Dpp signals via a receptor complex containing the type I receptors Tkv and/or Sax and the type II receptor Punt [13]. Activated Tkv/Sax phosphorylates the transcription factor Mad. Phosphorylated Mad binds the co-Smad Medea to regulate target gene expression. Repressive activity is conferred by binding of the Mad-Medea complex to silencer elements, which allow recruitment of the corepressor Schnurri [14]. Independent of the work described here, we carried out an in silico screen to identify transcription factors responsible for coordinated gene regulation following immune activation. This identified *Mad-Med-shn* silencer elements near many antimicrobial peptide (AMP) genes (Figure S2A). Notably, the silencer elements identified near *Defensin* are functional, repressing *pentagone/magu*, the surrounding gene [15]. This suggested that wound-induced Dpp might repress AMP expression via Mad.

**Figure 2:**
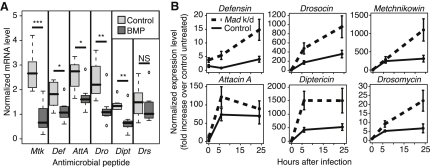
*dpp* Suppresses AMP Expression (A) AMP expression following wounding in flies overexpressing Dpp and Gbb under the control of heat-shock Gal4 (*tubulin-Gal80^ts^*/UAS-*gbb;hs-Gal4*/UAS-*dpp*) and in driver-only controls (*tubulin-Gal80^ts^*/+*;hs-Gal4*/+). Flies were heat shocked for 30 min beginning 30 min after wounding, and RNA samples were collected 3 hr after wounding. (B) AMP expression in *Mad* knockdown flies (*w^1118^;*UAS-*Mad-*IR/c564) and driver-only controls (*w^1118^;*c564/+) following PBS injection. Means are shown ±SEM. Expression is normalized to untreated driver-only controls. Assays were performed by qRT-PCR, and expression was initially normalized to *Rpl1*. ^∗∗∗^p < 0.001, ^∗∗^p < 0.01, ^∗^p < 0.05 by Mann-Whitney test. See also Figure S2.

To test whether Dpp is sufficient to repress AMP expression, we injected wild-type flies with human BMP-4, the homolog of *dpp*, or with vehicle only and assayed AMP responses to this injection. Each AMP assayed showed a lower transcript level in samples that had received BMP-4, relative to vehicle controls (Figure S2B). We then confirmed this result with the endogenous signal. Because BMPs often signal as heterodimers and, in these cases, the heterodimer is generally more potent [16], we overexpressed both *dpp* and *gbb* (the second BMP in the adult fly) in wounded animals under the control of heat-shock Gal4 (Figure S2C) and assayed AMP expression 3 hr after wounding. BMP expression induced following wounding reduced expression of five of six assayed AMPs (Figure 2A). Finally, to test the in vivo role of the *dpp-Mad* signal, we assayed AMP expression in flies with *Mad* knocked down in the fat body, the tissue primarily responsible for AMP expression upon systemic immune challenge. Loss of fat body *Mad* increased AMP expression, particularly after sterile wounding (Figure 2B).

These data indicate that Dpp represses AMP expression following wounding, particularly in the absence of infection. The presence of *Mad-Med-shn* silencer elements near AMP genes suggests that this repression is in part direct. Dpp may thus be important following tissue damage in the absence of infection to avoid unnecessary AMP responses.

### Dawdle Suppresses Melanization via the Activin Pathway

**Figure 3:**
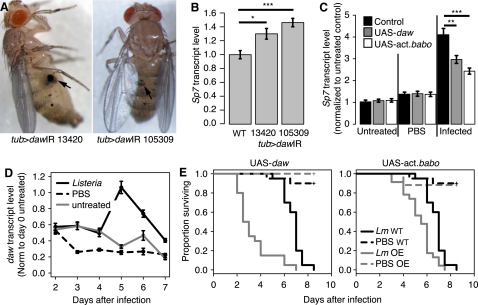
Dawdle Signals via the Activin Pathway to Suppress Melanization (A) Melanotic tumors in *daw* knockdown flies (*w;*UAS-*daw-*IR/+*;tubulin*-*Gal4*/+). Two independent inverted repeat (IR) lines are shown (VDRC13420 and VDRC105309). Tumors are indicated by arrows. (B) *Sp7* expression 5 days after eclosion in *daw* knockdowns and driver-only controls. (C) *Sp7* expression in flies overexpressing *daw* or activated *babo* in adult fat body (*w;*UAS-*daw*/c564*;tubulin*-*Gal80^ts^*/+ or c564/+*;*UAS-act.*babo*/*tubulin*-Gal80^ts^) relative to controls (c564/+*;tubulin*-Gal80^ts^/+). Animals were untreated or collected 6 hr postinjection with PBS or mixed *E. coli* and *M. luteus*. Expression is normalized to untreated driver-only controls. (D) *daw* expression following *Listeria* infection of wild-type flies. Expression is normalized to day 0 untreated levels. (E) Survival of *daw*- and activated *babo*-expressing flies following *Listeria* infection, relative to driver-only controls. (Lines labeled “OE” correspond to overexpressors.) Survival of both misexpression lines is different from controls (p < 0.001). For qRT-PCR assays in (B)–(D), expression was initially normalized to *Rpl1*, and means are shown ±SEM. ^∗∗∗^p < 0.001, ^∗∗^p < 0.01, ^∗^p < 0.05 by Mann-Whitney test. See also Figure S3.

To identify *daw*'s immune role, we produced animals carrying a ubiquitous *daw* knockdown (Figure S3A). Over 50% of these flies had melanotic tumors (Figure 3A), suggesting that *daw* inhibits melanization, a key effector mechanism of arthropod immunity. The melanization cascade is tightly controlled, presumably to prevent immune-induced pathology [17, 18]. On assaying known regulators of melanization in these flies, we found increased expression of *Serine protease 7* (*Sp7*), which is required specifically for infection-induced melanization [19] (Figure 3B).

*daw* signals primarily via the sole *Drosophila* type I activin receptor, Baboon (*babo*) [20]. To test whether *daw*-*babo* signaling is sufficient to inhibit *Sp7* expression, we assayed *Sp7* levels in flies overexpressing *daw* or activated *babo* (Figure S3B) in adult fat body using the Gal4 driver c564 with *tubulin*-Gal80^ts^. *daw* overexpression or activated *babo* expression dampened *Sp7* induction by infection but did not affect *Sp7* in untreated or PBS-injected animals (Figure 3C). Thus, endogenous *daw* inhibits *Sp7* expression in the absence of infection, whereas *daw* overexpression can inhibit infection-induced expression. In the context of our data on *daw* regulation, this suggests that activated *Dif* drives *daw* expression, shutting down *Sp7* to limit infection-induced melanization.

*Sp7* is important for resistance to *Listeria monocytogenes* and *Salmonella typhimurium* infections [21]. We thus examined the role of *daw* during *Listeria* infection. *Sp7* expression was induced early following *Listeria* infection (Figure S3D). The temperature shift involved in our infection protocol confounded interpretation of *daw* expression at early time points; however, *daw* was strongly induced on the fourth day postinfection, relative to untreated and PBS-injected controls (Figure 3D). The peak in *daw* expression 5 days postinfection (Figure 3D) correlated with a plateau in *Sp7* levels (Figure S3D); after this time, *daw* levels fell but *Sp7* did not change, implying the presence of other *Sp7* regulators. Overexpressing *daw* or activated *babo* resulted in rapid death from *Listeria* infection (Figure 3E), suggesting that suppression of *Sp7* by *daw* is detrimental to survival, much like the loss of *Sp7* through mutation [21].

Unlike BMP manipulations, activated *babo* expression in the fat body gave no consistent effect on AMPs (Figure S3C), and *Mad* knockdown did not induce melanization (data not shown). This indicates distinct immune roles for *daw* and *dpp*.

### *dpp* and *daw* Are Expressed in Subsets of Hemocytes

To identify signal-expressing tissues, we used *dpp.*blk1.40C.6-*Gal4* and *daw*^NP4661^ to drive expression of red fluorescent protein (mRFP). *dpp.*blk1.40C.6-Gal4 recapitulates *dpp* expression in most developmental contexts [22]; *daw*^NP4661^ places Gal4 in the endogenous *daw* locus.

In untreated animals, both Gal4 lines drove mRFP in a pattern similar to those seen with the hemocyte markers *Hemolectin* (*Hml*) and *croquemort* (*crq*) (Figures S4A–S4C) [23, 24]. However, unlike *Hml* and *crq*, *dpp*- and *daw*-expressing cells were most abundant in the thorax and around the dorsal vessel in the abdomen, with few cells visible in other abdominal regions or the legs (Figure S4C). *dpp*-Gal4 and *daw*-Gal4 were also expressed in pericardial nephrocytes, as was *crq*-Gal4 (Figure S4A), and in the adult salivary gland (Figure S4D). *dpp*-Gal4 was expressed in gut as previously described [25], whereas *daw*-Gal4 gave no gut expression (Figure S4E). These patterns were corroborated with different *dpp*-Gal4 and *daw*-Gal4 lines (data not shown). We detected no change in mRFP expression after infection or wounding with either driver (data not shown).

*Drosophila* hemocytes are macrophage-like cells that phagocytose bacteria and apoptotic cells and secrete extracellular matrix components and immune peptides [2]. Due to the hemocyte-like expression patterns of the *dpp*-Gal4 and *daw*-Gal4 lines, we assayed the phagocytic activity of *dpp*- and *daw*-expressing cells by injecting fluorescent dead *Staphylococcus aureus*, labeling phagocytes throughout the animal. All images were taken between 40 and 50 min after injection to prevent injection-induced changes in mRFP expression.

**Figure 4:**
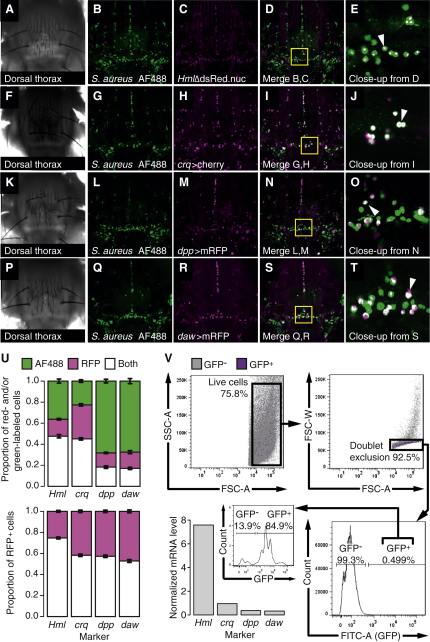
*dpp* and *dawdle* Are Expressed in Subsets of Phagocytes (A–T) Dorsal thoraxes of flies injected with Alexa Fluor 488-labeled *S. aureus*. (A–E) *Hml*Δ-*dsRed* flies (*w;Hml*ΔdsRed.nuc). (F–J) *crq*>*cherry* flies (*w^1118^;;crq*-*Gal4,*UAS-*mCD8-cherry*/+). (K–O) *dpp*>*mRFP* flies (*w;*UAS-*myr.mRFP*/+*;dpp.*blk1.40C.6-*Gal4*/+). (P–T) *daw*>*mRFP* flies (*w;*UAS-*myr.mRFP*/*daw*^NP4661^). (A, F, K, and P) Bright field of the region imaged in each row. (B, G, L, and Q) Maximum projection, Alexa Fluor 488 *S. aureus*-labeled phagocytes (green). (C, H, M, and R) Maximum projection, RFP (magenta). (D, I, N, and S) Merge of red and green showing overlap (white) between Alexa Fluor 488 *S. aureus* and RFP. (E, J, O, and T) Close-up of the indicated region of the previous image (yellow square), taken from a single focal plane to clarify the overlap between *S. aureus* and marker expression. Example double-labeled cells are indicated with white arrowheads. (U) Proportions of cells showing green fluorescence (Alexa Fluor 488 *S. aureus*), red fluorescence (RFP), or both. Top: these subsets as proportions of the total number of labeled cells. Bottom: proportions of RFP^+^ cells that were also AF488^+^/AF488^−^. Counts were taken from single images covering the entire dorsal thorax and abdomen. Full counts are given in Table S1. (V) Top and bottom right: fluorescence-activated cell sorting strategy for cells marked by *Hml*Δ>*eGFP* (*w^1118^;Hml*Δ*-Gal4,UAS-2xeGFP*/+). Gating (clockwise from top left): debris exclusion, doublet exclusion, eGFP expression. Bottom left: expression of *Hml*, *crq*, *dpp*, and *daw* in this sample (20,000 GFP^+^ cells) and purity analysis for this sample. See also Figure S4 and Table S1.

We then examined colocalization of fluorescent *S. aureus* with *Hml*, *crq*, *dpp*, and *daw* by confocal imaging on live adult flies (Figures 4A–4U). 74% of *Hml*^+^, 58% of *crq*^+^, 57% of *dpp*^+^, and 52% of *daw*^+^ cells phagocytosed detectable amounts of *S. aureus*. Conversely, 57% of phagocytic cells were *Hml*^+^, 67% *crq*^+^, 21% *dpp*^+^, and 20% *daw*^+^. The number of *dpp/daw*-negative phagocytes was thus much higher than the number of *Hml/crq*-negative phagocytes, confirming that only a fraction of phagocytes express *daw* or *dpp*. The apparent difference in phagocytic activity between *Hml*- and *crq*-expressing hemocytes, and the presence of *dpp*^+^, *dpp*^−^, *daw*^+^, and *daw*^−^ hemocytes, illustrates the importance of further characterization of the adult hemocyte population: both factors indicate that the adult hemocyte population consists of physiologically distinct subsets of cells.

To assess whether the numbers of hemocytes expressing *dpp* or *daw* changes following infection or wounding, we injected *dpp*>mRFP or *daw*>mRFP flies with PBS or *M. luteus*, waited 16 hr, and then counted mRFP-expressing cells. Neither injection changed the number of cells expressing *dpp*-Gal4 or *daw*-Gal4 (Table S1).

Finally, we confirmed that *dpp* and *daw* were expressed by *Hml*^+^ cells. We used fluorescence-activated cell sorting (FACS) to isolate *Hml*^+^ cells from adult *Drosophila*, an approach previously used in larvae [26] (Figure 4V). Quantitative RT-PCR on FACS-isolated hemocytes showed expression of *Hml*, as expected, as well as *crq*, *dpp*, and *daw* (Figure 4V; Figure S4F).

*dpp* and *daw* are therefore each expressed in a subset of hemocytes. Further work will be necessary to characterize the subsets of the adult hemocyte population, the extent to which these subsets overlap, and the implications of distinct gene expression profiles for hemocyte function.

### Conclusions

We show that the TGF-β superfamily members *daw* and *dpp* are physiological regulators of *Drosophila* immunity. *dpp* is induced by wounding and infection and helps resolve the antimicrobial peptide response, whereas *daw* is repressed by wounding, is induced by the *Toll* pathway, and limits infection-induced melanization. The modulation of the downstream signaling pathways in the fat body is sufficient to produce significant changes in whole-animal levels of target gene transcripts. However, although the fat body is responsible for the majority of induced AMP expression, many other tissues respond to immune activation. *dpp* and *daw*, as secreted signals, may act systemically to regulate other target genes in other tissues throughout the animal.

*dpp* and *daw* are expressed in hemocytes but also in other tissues. The regulation of these signals in a given tissue may reflect a distinct function for that tissue in sensing infection or wounding. The expression of *dpp* and *daw* in a fraction of hemocytes is particularly intriguing in this context. The fact that hemocyte-specific overexpression of *dpp* is sufficient to repress AMP induction [27] supports a hemocyte origin for this signal. Several types of hemocyte have been characterized in *Drosophila* larvae [3, 28]; however, the hemocytes of the adult fly have been largely neglected and are widely believed to consist of a single cell type. To our knowledge, this is the first indication that the hemocyte population in the adult fly is comprised of distinct subsets of cells that can be defined through distinct gene expression profiles. We think it likely that expression of *dpp* and *daw* by a subset or subsets of phagocytes indicates distinct immunomodulatory functions for these cells.

Both *dpp* and *daw* inhibit immune responses. This aligns the fly with mammals, in which both activin/TGF-β-like and BMP-like signals are broadly anti-inflammatory [29–31], in contrast with *C. elegans*, where the TGF-β superfamily member *dbl-1* promotes a variety of antimicrobial responses [32, 33]. Study of these signals in *Drosophila* will allow further characterization of individual signals and of mechanisms of signal integration in a way that is not currently possible in more complex systems.

## Supplemental Information

Document S1. Four Figures, One Table, and Supplemental Experimental Procedures
