# Supplementary material for: Multiple TGF-β Superfamily Signals Modulate the Adult Drosophila Immune Response
Source: Curr Biol. 2011 Oct 11;21(19):1672–7. doi: 10.1016/j.cub.2011.08.048 (PMC3191266; doi:10.1016/j.cub.2011.08.048)
Supplement: Document S1. Four Figures, One Table, and Supplemental Experimental Procedures [file mmc1.pdf]

Current Biology, Volume *21*

Supplemental Information

Multiple TGF-β Superfamily Signals Modulate the Adult *Drosophila* Immune Response

Rebecca I. Clark, Katie J. Woodcock, Frédéric Geissmann, Céline Trouillet, and Marc S. Dionne

Supplemental Inventory

Figure S1, Related to Figure 1
Shows regulation of *dpp* and *daw* following injection with *E coli*, as an addition to the regulation by *M luteus* injection shown in Figure 1; regulation of *daw* in *Rel* mutants; induction of *Drs* by UAS-Tl^{10b}; and regulation of *dpp* in *Tak1* mutants.

Figure S2, Related to Figure 2
Shows *Mad-Med-shn* binding sites in AMP genomic regions, suggesting that the *dpp* signal may regulate AMP expression. Also shows the regulation of AMP expression by injection of the *dpp* homolog BMP-4, and the adult-specific over-expression of *dpp* and *gbb*. Both supporting the conclusion that the *dpp* signal regulates AMP expression, and in corroboration of methods used in Figure 2.

Figure S3, Related to Figure 3
Shows expression levels of *daw* and *babo* in experimental animals, to corroborate methods used in Figure 3. Also shows AMP expression in *daw* knockdowns and expression of *Sp7* following *Listeria* infection.

Figure S4, Related to Figure 4
Shows anatomical survey of *dpp* and *daw* expression in uninfected animals, RT-PCR results from an independent repeat of the FACS experiment in Figure 4, and results from sorting on non-GFP-expressing flies.

Table S1, Related to Figure 4
Shows full cell counts from which quantitative analysis in figure 4U is derived.

Supplemental Experimental Procedures

Supplemental References

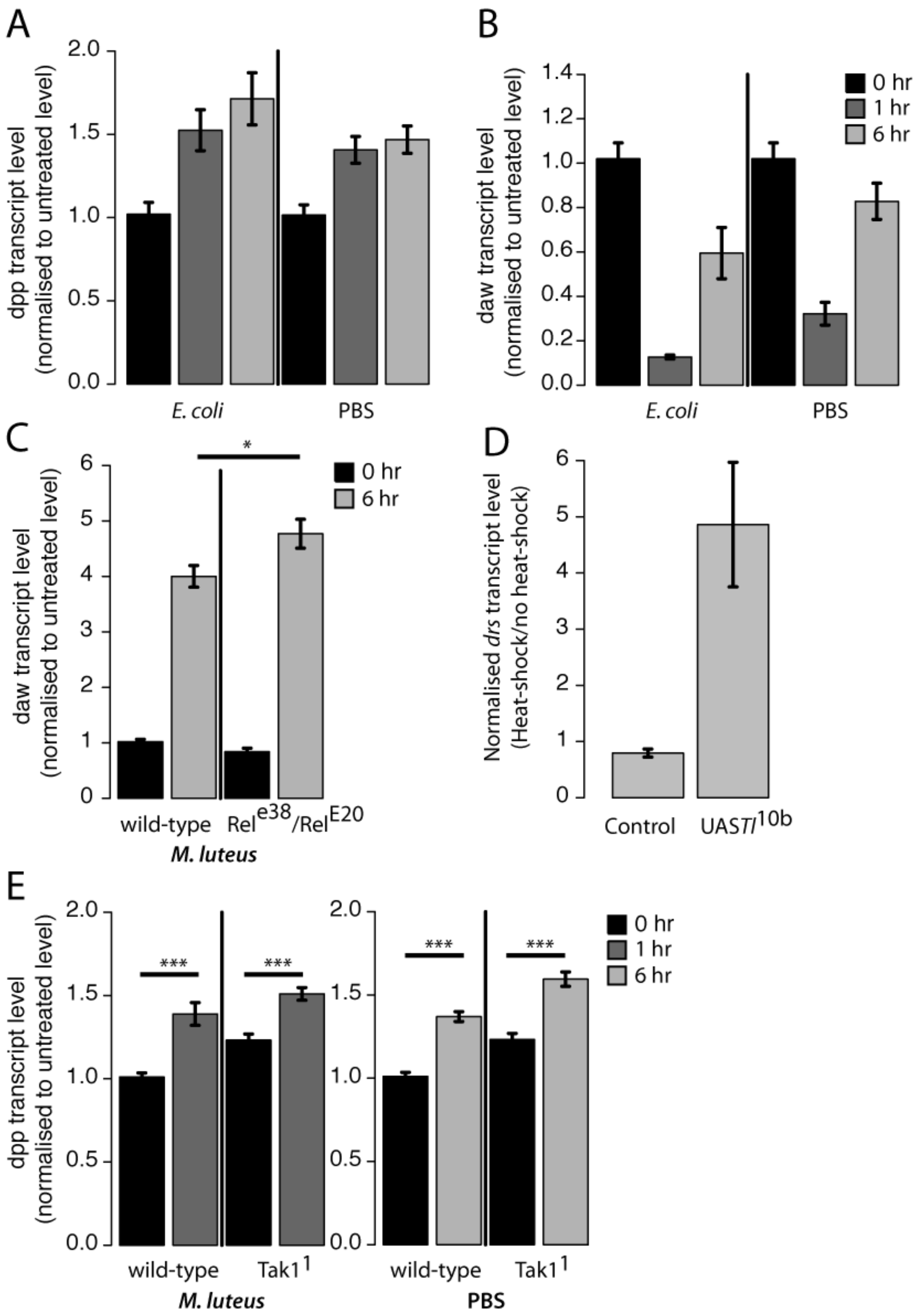
A
dpp transcript level (normalised to untreated level)
E. coli
PBS
B
daw transcript level (normalised to untreated level)
0 hr
1 hr
6 hr
E. coli
PBS
C
daw transcript level (normalised to untreated level)
0 hr
6 hr
*
wild-type
Rele38/RelE20
M. luteus
D
Normalised drs transcript level (Heat-shock/no heat-shock)
Control
UASTl10b
E
dpp transcript level (normalised to untreated level)
0 hr
1 hr
6 hr
***
wild-type
Tak11
M. luteus
PBS

Figure S1.

Figure S1. Regulation of *dpp* and *daw*, Related to Figure 1

(A) *dpp* expression in Oregon-R wild-type flies following *E. coli* infection or PBS injection, normalized to untreated controls.

(B) *daw* expression in Oregon-R wild-type flies following *E. coli* infection or PBS injection, normalized to untreated controls.

(C) *daw* expression in untreated and *M. luteus* infected flies mutant for the NFκB transcription factor *rel* (;;Rel^{E20}/Rel^{e38}), and wild-type controls, normalized to untreated wild-type controls.

(D) *Drs* expression level three hours after heat-shock treatment in flies carrying UAS*Tl10b* [1] (UASTl^{10b}.myc/*tubulin*-Gal80^{ts};*heat-shock*-Gal4/+**)**, an activated allele of *Toll*, and driver only controls (*tubulin*-Gal80^{ts}/+;*heat-shock*-Gal4/+); done to test activation of the pathway (*Drs* is a direct and specific *Toll* target). Expression levels after heat-shock are normalized to non-heat-shocked controls of the same genotype.

(E) *dpp* expression in wild-type control animals and *Tak1* mutants ($Tak1^{1}$;) 1 hour following *M. luteus* injection, and 6 hours following PBS injection. Expression level is normalized to untreated wild-type controls. Wild-type controls are Oregon-R throughout. Assays were done by qRT-PCR and expression initially normalized to *Rpl1* levels as a loading control. Means are shown +/- SEM. ***p < 0.001, **p < 0.01, *p < 0.05.

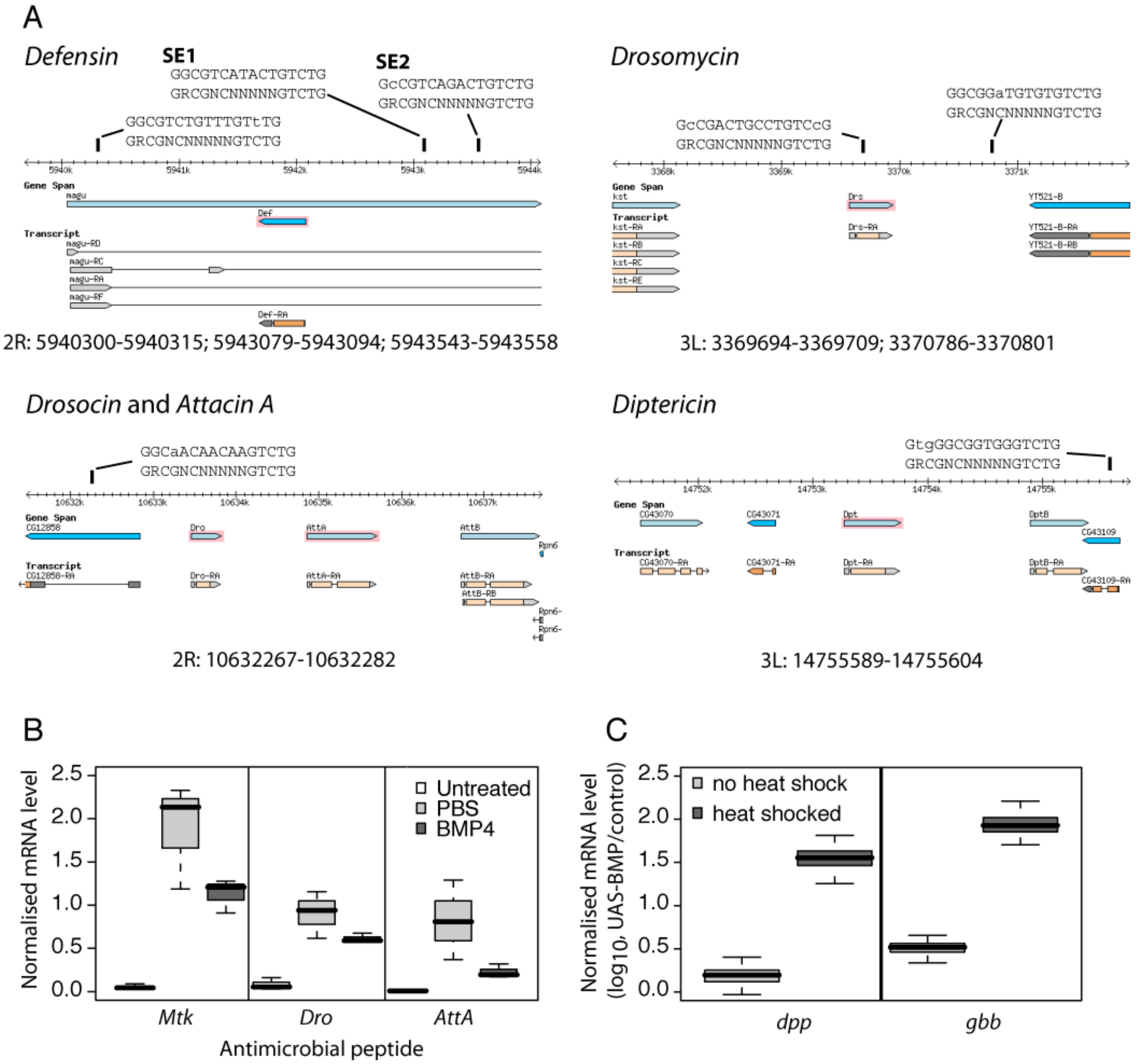

Figure S2. Regulation of AMP Expression by *dpp*, Related to Figure 2

(A) AMP genomic regions contain Mad-Med-Shn binding sites. The gene model for each of five AMPs is shown. *Metchnikowin* is not shown because it has no high-scoring Mad-Med-Shn sites. Vertical black lines show the locations of putative Mad-Med-Shn binding sites; the precise positions of these sites are indicated below each diagram (coordinates are from version 5 of the *Drosophila* genome sequence). The sequence of each site is shown; lowercase indicates nucleotides divergent from the notional ideal (GRCGNCNNNNNGTCTG) [2, 3]. Sequence data is shown in lower case where it deviates from the ideal site. SE1 and SE2 from Vuilleumier et al (2010) are marked on the *Defensin* gene model [4].

(B) Injection of human BMP-4 suppresses the AMP response to wounding. Transcript levels (normalized to the loading control *Rpl1*) are shown in untreated, vehicle injected, and human BMP-4 injected samples, six hours postinjection.

(C) Adult-specific over-expression of BMP ligands. Transcript levels of *dpp* and *gbb* are shown as a ratio of levels in flies over-expressing both ligands under the control of a heat-shock Gal4 (*tubulin*-Gal80*ts*/UAS*gbb*; *heat-shock*-Gal4/UAS*dpp*) and levels in driver only controls (*tubulin*-Gal80*ts*/+; *heat-shock*-Gal4/+). Ratios of transcript levels in no heat-shock controls are also shown. Transcript level was initially normalized to *Rpl1* levels as a loading control.

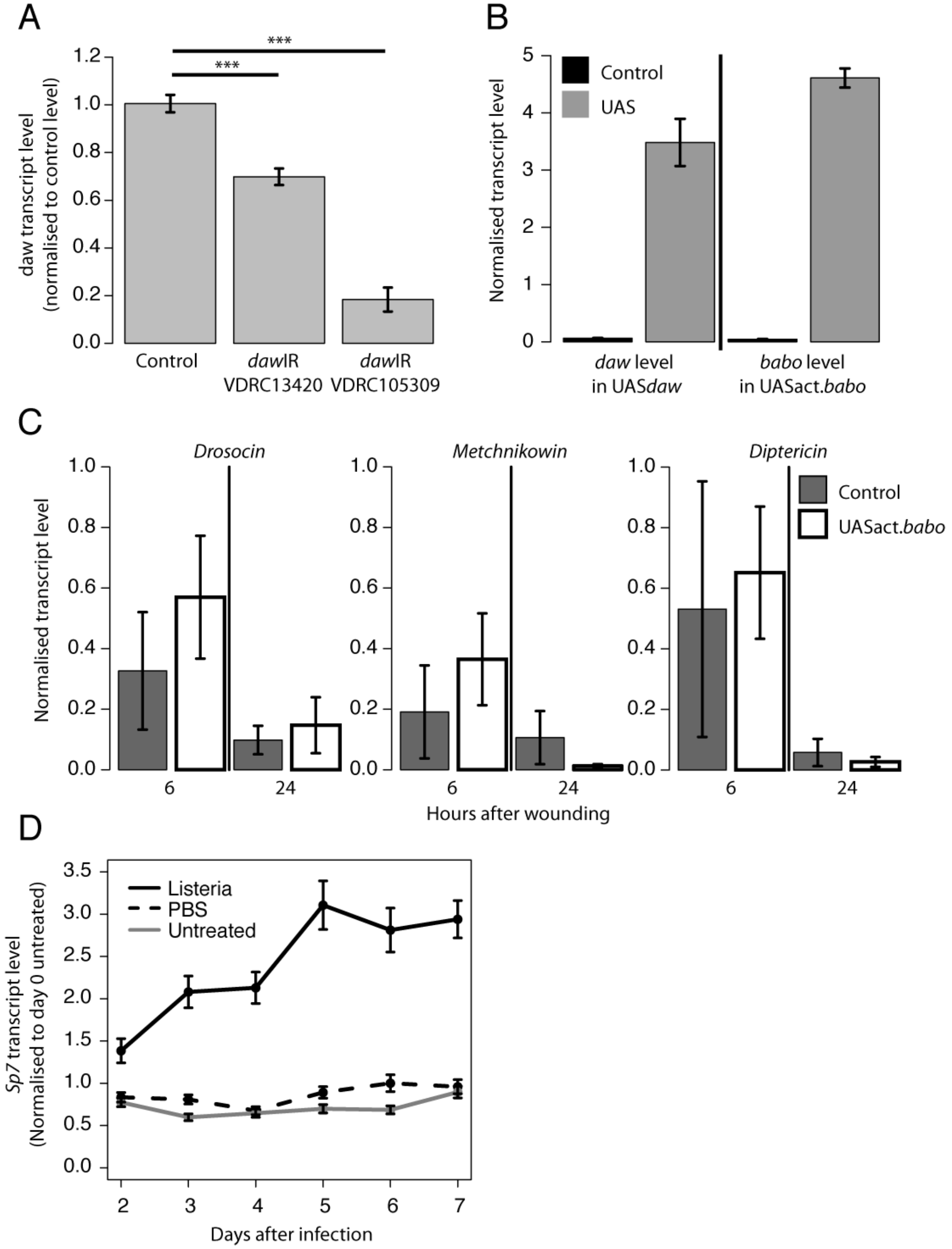

Figure S3. Dawdle Signals via the Activin Pathway to Suppress Melanization, Related to Figure 3

(A) Transcript levels of *daw* are shown in animals carrying UAS*daw* inverted repeats under the control of the ubiquitous driver *tubulin*-Gal4 (w; UAS*daw*IR/+; *tubulin*-Gal4/+), compared to driver only controls (w;; *tubulin*-Gal4/+). Transcript levels are shown from animals carrying two independent IR lines (VDRC13420 and VDRC105309).

(B) Transcript levels of *daw* and *babo* in flies carrying UAS*daw* or UASact.*babo* under the control of the fat body specific driver c564 (c564/UAS*daw*; *tubulin*-Gal80^{ts}/+ or c564/+; *tubulin*-Gal80^{ts}/UASact.*babo*), and driver only controls (c564/+; *tubulin*-Gal80^{ts}/+). Samples were taken following overnight incubation at 29°C to inactivate the temperature sensitive allele of the Gal4 repressor Gal80, allowing induction of UAS driven expression by the Gal4.

(C) AMP expression in animals carrying UASact.*babo*, under the control of c564 (as in B), and driver only controls. Samples were taken at 6 and 24 hours following PBS injection.

(D) *Sp7* transcript levels following *Listeria monocytogenes* infection of Oregon-R wild-type flies. Transcript levels in infected animals, PBS injected and untreated animals are shown from two days postinjection, and are normalized to day 0 untreated levels. Transcript level was normalized to *Rpl1* levels as a loading control, and means are shown +/- SEM. ***p < 0.001, **p < 0.01, *p < 0.05.

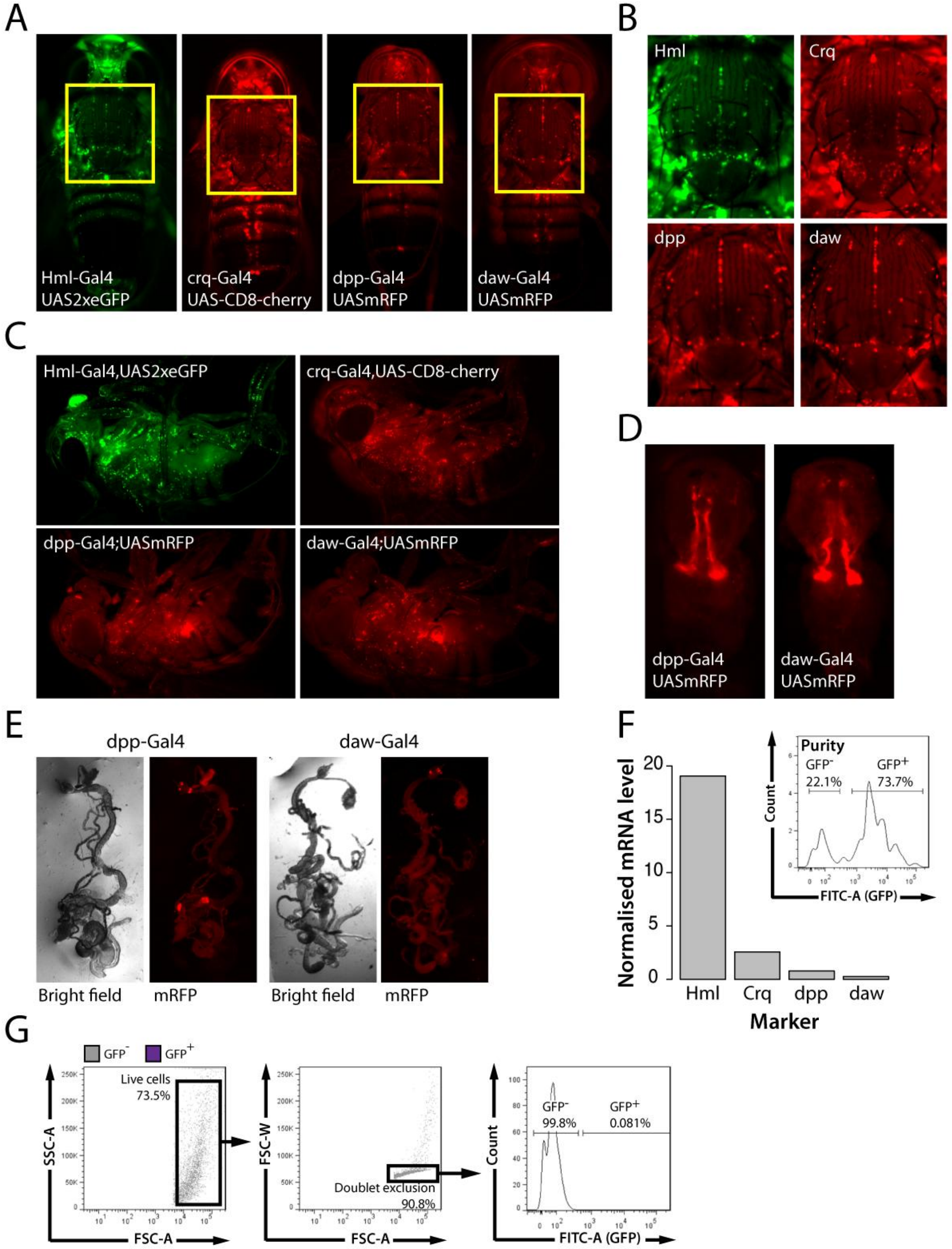
A
Hml-Gal4
UAS2xeGFP
crq-Gal4
UAS-CD8-cherry
dpp-Gal4
UASmRFP
daw-Gal4
UASmRFP
B
Hml
Crq
dpp
daw
C
Hml-Gal4,UAS2xeGFP
crq-Gal4,UAS-CD8-cherry
dpp-Gal4;UASmRFP
daw-Gal4;UASmRFP
D
dpp-Gal4
UASmRFP
daw-Gal4
UASmRFP
E
dpp-Gal4
daw-Gal4
Bright field
mRFP
Bright field
mRFP
F
Purity
GFP- 22.1%
GFP+ 73.7%
Count
FITC-A (GFP)
Normalised mRNA level
Hml
Crq
dpp
daw
Marker
G
GFP-
GFP+
Live cells
73.5%
SSC-A
FSC-A
FSC-W
Doublet exclusion
90.8%
FSC-A
GFP-
99.8%
GFP+
0.081%
Count
FITC-A (GFP)

Figure S4.

Figure S4. Tissue Localization of *dpp* and *daw*, Related to Figure 4

(A) A dorsal view of untreated animals, showing the location of expression of *Hml*-, *crq*-, *dpp*- and *daw*-Gal4s (*w*;*HmlΔ*-gal4,UAS-2xeGFP, w^{1118};;*crq*-Gal4,UAS-mCD8-cherry/+, *w*;UAS-myr.mRFP/+;*dpp*.blk-Gal4/+, *w*;UAS-myr.mRFP/daw^{NP4661}). Yellow boxes outline the region shown in B.

(B) Close-ups of the dorsal thorax region from each of the four images shown in A.

(C) A side view of untreated animals, showing the location of expression of *Hml*-, *crq*-, *dpp*- and *daw*-Gal4s.

(D) A ventral view of untreated animals carrying *dpp*- or *daw*-Gal4 driving mRFP. The ventral thoracic cuticle has been removed to show the salivary glands.

(E) Gut dissections from untreated animals carrying *dpp*- or *daw*-Gal4 driving mRFP. Both bright-field and fluorescent images are shown.

(F) Expression level of *Hml*, *crq*, *dpp* and *daw* in a FACS sample containing 18,599 GFP positive cells from w;*HmlΔ*-gal4,UAS-2xeGFP animals. The inset shows the purity analysis for this FACS sample, illustrating that 73.7% of cells sorted for purity analysis (390 events) were GFP positive.

(G) FACS analysis of the nonfluorescent control (from Oregon-R wild-type flies) for comparison with the sort shown in Figure 4V.

Table S1. Cell Counts Used to Produce Figure 4U

Location	Marker	Treatment	n	AF488	mRFP	Colocalized	Total
Thorax	*Hml*	Untreated	13	90.2 +/- 7.9	73.5 +/- 8.4	57.8 +/- 6.8	106 +/- 9.2
	crq	Untreated	10	58.8 +/- 4.0	64.6 +/- 3.7	47.1 +/- 3.4	76.3 +/- 4.2
	dpp	Untreated	9	68.8 +/- 3.16	37.3 +/- 1.9	22.8 +/- 1.8	83.3 +/- 3.5
		PBS	8	53.9 +/- 5.9	33.4 +/- 2.1	17.6 +/-1.56	69.6 +/-4.8
		M. luteus	8	63 +/- 2.9	36 +/- 1.9	20.1 +/- 1.8	78.9 +/- 3.2
	daw	Untreated	7	35.7 +/- 1.4	36.1 +/- 3.0	15.1 +/- 1.2	56.7 +/- 3.1
		PBS	7	34 +/- 2.4	38.3 +/- 3.2	15.9 +/- 2.0	56.4 +/- 3.5
		M. luteus	7	45.7 +/- 6.3	42.7 +/- 3.1	17.3 +/- 2.1	71.1 +/- 4.6
Abdomen	*Hml*	Untreated	13	206.1 +/- 13.3	152.9 +/- 11.6	110.8 +/- 7.5	248.2 +/- 17.3
	crq	Untreated	10	208.8 +/- 9.8	241.2 +/- 12.3	131.3 +/- 7.2	318.7 +/- 14.0
	dpp	Untreated	9	179.7 +/- 9.9	53.4 +/- 4.1	29.3 +/- 3.2	203.8 +/- 10.7
		PBS	8	169.9 +/- 12.8	53.9 +/- 9.3	30.3 +/- 5.6	193.5 +/- 15.7
		M. luteus	8	201.5 +/- 14.4	64.9 +/- 8.8	33.1 +/- 4.5	233.3 +/- 18.7
	daw	Untreated	7	150.6 +/- 11.0	35.9 +/- 4.7	22.7 +/- 3.0	163.7 +/- 12.1
		PBS	7	133.6 +/- 9.5	36.3 +/- 9.7	16.3 +/- 3.6	153.6 +/- 13.3
		M. luteus	7	148.7 +/- 12.2	34.1 +/- 6.5	13.6 +/- 1.8	169.3 +/- 15.6
Combined	*Hml*	Untreated	13	296.3 +/- 18.9	226.4 +/- 18.2	168.5 +/- 12.8	354.2 +/- 23.8
	crq	Untreated	10	267.6 +/- 12.4	305.8 +/- 14.0	178.4 +/- 9.5	395 +/- 15.9
	dpp	Untreated	9	248.4 +/- 10.1	90.8 +/- 5.2	52.1 +/- 3.4	287.1 +/- 11.3
		PBS	8	223.8 +/- 15.0	87.3 +/- 9.7	47.9 +/- 5.2	263.1 +/- 16.1
		M. luteus	8	264.5 +/- 16.1	100.9 +/- 9.7	53.3 +/- 6.0	312.1 +/- 19.7
	daw	Untreated	7	186.3 +/- 11.9	72 +/- 7.3	37.7 +/- 3.7	220.4 +/- 14.1
		PBS	7	167.6 +/- 11.3	74.6 +/- 11.6	32.1 +/- 5.5	210 +/- 15.1
		M. luteus	7	194.4 +/- 14.8	76.9 +/- 7.4	30.9 +/- 2.7	240.4 +/- 17.3

Also shows the cell counts from flies treated with an *M. luteus* infection or with a PBS injection that are not illustrated in the main figure. Counts are shown as the mean +/- SEM.

Supplemental Experimental Procedures

Fly Culture and Stocks

Flies were maintained on food containing 10% w/v Brewer's yeast, 8% fructose, 2% polenta and 0.8% Agar, at 25°C and 60% relative humidity unless otherwise stated. Adult males were collected soon after eclosion and transferred to fresh vials to age to 5-10 days old prior to treatment. Experimental and control flies carrying both the c564 driver and *tubulin*-Gal80^{ts} were raised at 18°C and transferred to 29°C to deactivate the Gal80 the evening prior to morning treatment. Experimental and control flies carrying both the *heat-shock*-Gal4 and *tubulin*-Gal80^{ts} were raised and maintained at 25°C, heat-shock was carried out 30 minutes after wounding and consisted of 30 minutes at 37°C in air. *HmlΔ-dsRed.nuc* was produced by cloning the HmlΔ promoter fragment into pRedH-Stinger and will be fully described elsewhere.

The *Mad* RNAi stock and *dawdle* RNAi stocks were obtained from the VDRC RNAi library (stock numbers 12635, 13420 and 105309). Heat-shock Gal4, Gal80^{ts}, c564, *dpp*-Gal4, and all wild-type lines were obtained from Bloomington. w^{1118};; *crq*-Gal4,UAS-mCD8-cherry/ TM6c,Sb^{1} flies were produced from commonly available components [5]. *w*;*HmlΔ*-gal4,UAS-2xeGFP flies were a gift from Sergey Sinenko [6]; UAS-activated *baboon*, UAS-*daw*, daw^{NP6274} and daw^{NP4661} were gifts of Julian Ng; *Dif* mutants were gifts of Dominique Ferrandon; *Rel* mutants were gifts from Dan Hultmark [7]; UAS-*gbb* was a gift of Robert Ray.

Bacterial Methods

L. monocytogenes (NCTC7973) was grown overnight in brain heart infusion medium, standing, at 37°C. *E. coli* and *M. luteus* were grown overnight in LB medium, shaking, at 37°C. Bacterial cultures were pelleted and resuspended in PBS. *L. monocytogenes* cultures were then adjusted to an OD600 of 0.1, and *E. coli* and *M. luteus* cultures to an OD600 of 1. Mixed septic infections were done with an equal mix of OD-adjusted *E. coli* and *M. luteus* cultures (retaining a final OD of 1).

Fly Injections and Wounding

Adult male flies, aged between 5 and 10 days, were injected with 50nl of the appropriate bacterial suspension, or sterile filtered PBS containing 0.2% tween-80, as previously described [8]. Injected flies were transferred to fresh vials and maintained at 29°C following *Listeria* infection, or for genotypes including the *tubulin*-Gal80^{ts}, and at 25°C following all other treatments. *Listeria* infected flies and their controls were anesthetized and transferred to fresh food at four days postinfection. To collect survival data dead flies were counted twice daily. Flies treated with Human recombinant BMP-4 (R&D Systems) were injected with 50nl of PBS with 0.1% Bovine Serum Albumin either with or without 0.25ng BMP-4 and maintained at 25°C.

Quantitative Real-Time PCR

RNA extractions were carried out in TRIzol (Invitrogen) following the manufacturer's directions. cDNA synthesis was carried out using the First Strand cDNA Synthesis Kit (Fermentas), priming with random hexamers. PCR was performed with Sensimix SYBR Green no-ROX (Bioline) on a Corbett Rotor-Gene 6000. The cycling conditions used throughout were as follows: Hold 95°C for 10 minutes, then 40 cycles of 95°C for 15s, 57°C for 30s, 72°C for 30s.

All calculated gene expression values were initially normalized to the value of the loading control gene, *Rpl1*, prior to further analysis. The primer sequences used in this study are as follows:

Gene	Left Primer	Right Primer
Rpl1	TCCACCTTGAAGAAGGGCTA	TTGCGGATCTCCTCAGACTT
Dro	CCATCGAGGATCACCTGACT	CTTTAGGCGGGCAGAATG
Mtk	TCTTGGAGCGATTTTTCTGG	TCTGCCAGCACTGATGTAGC
AttA	CACAATGTGGTGGGTCAGG	GGCACCATGACCAGCATT
Def	TTCTCGTGGCTATCGCTTTT	GGAGAGTAGGTCGCATGTGG
Dipt	ACCGCAGTACCCACTCAATC	CCCAAGTGCTGTCCATATCC
Drs	GTACTTGTTCGCCCTCTTCG	CTTGCACACACGACGACAG
dpp	CCTTGGAGCCTCTGTCGAT	TGCACTCTGATCTGGGATTTT
gbb	CCAGATGCAGACCCTGTACAT	CTGGTGCGATGATCCAGTC
daw	GGTGGATCAGCAGAAGGACT	GCCACTGATCCAGTGTTTGA
Sp7	AGTTGCGGTGGCAGTTTG	CCCAACCGAACGGTAGTTAG

Confocal Microscopy

Alexa Fluor 488 labelled *Staphylococcus aureus* (Invitrogen) was prepared according to manufacturer's instructions, and diluted 10-fold prior to injection. Flies were injected with 50nl (as described above) and imaged 40 minutes later. Flies were immobilized by CO_2 anesthetization for 10 minutes, then affixed to a coverslip using a small drop of super-glue, and imaged through the coverslip and glue. Flies were imaged on a Leica SP5 confocal microscope and images were processed using Fiji. Images for cell counts were taken at 10x, to include the dorsal thorax and abdomen in the same image. Cell counts were performed using the MATLAB spot function in Imaris x64 7.3.0. Counts were performed separately on the thoracic and abdominal regions. A threshold value for each region was set on a representative image of each genotype and was then kept consistent for all images of that genotype. Infected or wounded flies imaged for cell counts were treated with *M. luteus* or PBS injection (as described above) the evening prior to imaging. Flies were left at 25ºC overnight in order to allow sufficient time for the transcription and processing of mRFP, to ensure that mRFP expression would fully reflect the activity of the relevant Gal4.

Fluorescence-Activated Cell Sorting (FACS)

FACS samples were prepared from 100 flies expressing GFP under the control of HmlΔ-Gal4 (*w^{1118}; HmlΔ-gal4,UAS2xeGFP/+*), or from 100 Oregon-R wild-type flies as a nonfluorescent control. Anesthetized flies were mashed through a 70µM filter using ice-cold PBS containing 2mM EDTA as a buffer. Samples were then centrifuged at low speed (150g for 15min at 4º) to pellet intact cells. Pelleted material was then resuspended in ice-cold buffer and this wash step repeated. Samples were further filtered though 40µM filters prior to sorting. Cells were sorted by GFP expression on a BD Aria II cell sorter using a 100µM nozzle and 20psi pressure. An initial sort was carried out into PBS/EDTA, and this sample was re-analyzed for sample purity. The main sort was then carried out into the RLT buffer of the RNeasy Plus Micro kit (Qiagen), and this sample processed immediately for RNA extraction.

Supplemental References

1. Lemaitre, B., Meister, M., Govind, S., Georgel, P., Steward, R., Reichhart, J.M., and Hoffmann, J.A. (1995). Functional analysis and regulation of nuclear import of dorsal during the immune response in Drosophila. Embo J *14*, 536-545.
2. Pyrowolakis, G., Hartmann, B., Muller, B., Basler, K., and Affolter, M. (2004). A simple molecular complex mediates widespread BMP-induced repression during Drosophila development. Dev Cell *7*, 229-240.
3. Yao, L.C., Phin, S., Cho, J., Rushlow, C., Arora, K., and Warrior, R. (2008). Multiple modular promoter elements drive graded brinker expression in response to the Dpp morphogen gradient. Development *135*, 2183-2192.
4. Vuilleumier, R., Springhorn, A., Patterson, L., Koidl, S., Hammerschmidt, M., Affolter, M., and Pyrowolakis, G. (2010). Control of Dpp morphogen signalling by a secreted feedback regulator. Nat Cell Biol *12*, 611-617.
5. Olofsson, B., and Page, D.T. (2005). Condensation of the central nervous system in embryonic Drosophila is inhibited by blocking hemocyte migration or neural activity. Dev Biol *279*, 233-243.
6. Sinenko, S.A., and Mathey-Prevot, B. (2004). Increased expression of Drosophila tetraspanin, Tsp68C, suppresses the abnormal proliferation of ytr-deficient and Ras/Raf-activated hemocytes. Oncogene *23*, 9120-9128.
7. Hedengren, M., Asling, B., Dushay, M.S., Ando, I., Ekengren, S., Wihlborg, M., and Hultmark, D. (1999). Relish, a central factor in the control of humoral but not cellular immunity in Drosophila. Mol Cell *4*, 827-837.
8. Dionne, M.S., Ghori, N., and Schneider, D.S. (2003). Drosophila melanogaster is a genetically tractable model host for Mycobacterium marinum. Infect Immun *71*, 3540-3550.
